# Anticancer activity of *Astragalus ovinus* against 7, 12 dimethyl benz (a) anthracene (DMBA)-induced breast cancer in rats

**Published:** 2020

**Authors:** Fouad Mehraban, Mostafa Mostafazadeh, Hossein Sadeghi, Arsalan Azizi, Mehdi Akbartabar Toori, Bita Gramizadeh, Vahid Barati, Heibatollah Sadeghi

**Affiliations:** 1 *Institute of Biochemistry and Biophysics (IBB), Tehran University, Tehran, Iran*; 2 *Department of Biochemistry and Clinical Laboratories, Tabriz University of Medical Sciences, Tabriz, Iran*; 3 *Medicinal Plants Research Center, Yasuj University of Medical Sciences, Yasuj, Iran*; 4 *Department of Pathology, Faculty of Medicine, Yasuj University of Medical Sciences, Yasuj, Iran*; 5 *Social Determinants of Health Research Center, Yasuj University of Medical Sciences, Yasuj, Iran*; 6 *Department of Pathology, Faculty of Medicine, Shiraz University of Medical Sciences, Shiraz, Iran*; 7 *Cellular and Molecular Research Center, Faculty of Medicine, Yasuj University of Medical Sciences, Yasuj, Iran*; 8 *Department of Biochemistry, Faculty of Medicine, Yasuj University of Medical Sciences, Yasuj, Iran*

**Keywords:** Antioxidant, Astragalus ovinous, Breast cancer, Dimethyl-1, 2-benzanthracene (DMBA), Rat

## Abstract

**Objective:**

Some species of *Astragalus* are used for the treatment of various types of cancer. The present study was designed to evaluate the anticancer potential of *Astragalus ovinus* extract (AOE) against DMBA-induced breast carcinoma in rats.

**Materials and Methods:**

The anti-tumor and antioxidant effects of AOE were evaluated against DMBA-induced breast carcinoma in rats using DPPH, FRAP and ABTS technique, respectively. Forty adult female Sprague-Dawley rats were randomly divided into four groups including the control group received a single dose of DMBA solvent orally, and groups II, III and IV received a single dose of DMBA (40 mg/kg) dissolved in olive oil. Groups I and II received normal saline and groups III and IV were treated with AOE orally (120 and 240 mg/kg respectively) for 60 consecutive days. Chemopreventive effects were assessed in terms of diameter and volume of tumors, expression levels of PCNA, and serum levels of CA15.3, p53, MDA, CAT, and calcium, and histopathological features

**Results:**

AOE contained a noticeable amount of phenolic and flavonoids compounds. This extract showed a potent antioxidant activity both *in vitro* and *in vivo*. AOE significantly decreased the diameter and volume of tumors (p<0.01) and reduced the serum levels of CA15.3 (p<0.001), p53 (p<0.01), MDA (p<0.001), and calcium (p<0.01). AOE also decreased the expression of PCNA in cancerous tissues and reduced the histopathological deformity.

**Conclusion:**

According to the data, AOE produced a significant chemopreventive activity in DMBA-induced breast tumors in rats, probably due to its antioxidant and its inhibitory effect on some tumorigenicity markers such as CA15.3, p53 and PCNA activity.

## Introduction

Breast cancer (BC) is the most common cancer among women in the United States with an estimated for 30% of all new cancer diagnoses in women (Siegel et al., 2019 ▶ ; Ferlay et al., 2019 ▶ ). This cancer begins in the breast and metastasizes to other tissues such as the lymph nodes, lungs, liver, bones and brain (Ferlay et al., 2015 ▶). 

Nowadays, surgery, chemotherapy, and radiation therapy, or a combination of them are the main options for treatment of BC. Despite these therapeutic options, the mortality rate of BC remains high (Alawode, 2013 ▶; Nelson et al., 2014 ▶). In the last decades, an increasing body of evidence indicated the ability of natural products to regulate signaling pathways of apoptosis as an anti-cancer agent. In this regard, herbal medicine plays an important role in the finding and the evolution of numerous agents for the management of different types of lethal diseases including cancer (Morse et al., 2000 ▶; Dias et al., 2012 ▶). 

*Astragalus* (Fabaceae) is a large genus and is native to temperate regions of the northern hemisphere. Several important pharmacologically active components including polysaccharides, saponins and phenolics and some toxic compounds such as the indolizidine alkaloids, aliphatic nitro compounds and seleniferous derivatives were identified in *Astragalus *genus (Mehraban et al., 2014 ▶). In Chinese traditional medicine, *Astragalus* is recommended as an anti-cancer treatment (Block and Mead, 2003 ▶; Zheng et al., 2019 ▶). Furthermore, immunomodulatory and anti-cancer activity of *Astragalus* genus were reported in several studies (Wang et al., 2015 ▶; Zhu et al., 2015 ▶; Li W et al., 2020 ▶; Zhou et al., 2018 ▶; Zhou., et al. 2018 ▶).

In recent years, some species of *Astragalus* such as *Astragalus membranaceus* and *Astragalus vogelii *were investigated in treating various cancers such as BC (Zhou et al., 2018 ▶; Al-Harbi et al., 2014 ▶; Liu et al., 2019 ▶). To our knowledge, there is no study about the anticancer activity of *A. ovinus*. So, the main purpose of this research was to investigate the anticancer properties of *Astragalus ovinus* extract (AOE) against 7, 12 dimethyl benz (a) anthracene (DMBA)-induced breast cancer in rats, with a particular focus on pathways involved in BC, including the antioxidant-oxidant balance: malondialdehyde (MDA), catalase (CAT), proliferating cell nuclear antigen (PCNA) and extracellular calcium, apoptotic (P53/TP53) and metastatic factors (CA15.3). 

## Materials and Methods

**Chemicals**

2, 2-Diphenyl-1-picrylhydrazyl (DPPH), 2, 2′azinobis-(3-ethylbenzthiazoline-6-sulphonic acid) (ABTS), 2, 4, 6-tri(2-pyridyl)-striazine (TPTZ), Folin-Ciocalteau reagent, gallic acid, 7, 12-dimethylbenz[a]anthracene (DMBA), 1, 1, 3, 3-tetraethoxypropan (TEP), trichloroacetic acid (TCA), and thiobarbituric acid (TBA) were obtained from Sigma–Aldrich Chemical Co (St. Louis, MO). Rat Mammary Carcinoma Marker/Carbohydrate Antigen 15-3 (CA15-3) and Rat P53/Tumor Protein (P53/TP53) ELISA kits were purchased from Novateinbio Biosciences, Immunohistochemistry (IHC) kit from Abcam Company (United Kingdom), DAB from Dako (Denmark), Envision secondary antibody from BioGenex and calcium kit from Dermankav (Iran). All other chemicals were of analytical grade.

**Plant materials**

Leaves of *A. ovinus* were collected from the Kakan suburbs of Yasuj (Kohgiluyeh va Boyer-Ahmad province, Iran) at the end of spring 2014 and identified by Dr A. Jafari (Department of Botany, Yasuj University, Yasuj, Iran) and a voucher specimen (Herbarium No. HYU30854) was deposited there. The leaves of the plant were air-dried and protected from direct sunlight.

**Preparation of hydroalcoholic extract**

The powdered leaves (200 g) were extracted two-times using l liter mixture of ethanol: water (70:30) at 45^◦^C for 48 hr. The extract was filtered and the organic solvent was completely removed under reduced pressure in a rotary evaporator at 60^◦^C and finally, freeze-dried to get AOE. The extract was collected and sealed for next use (Sadeghi et al., 2014 ▶; Panahi et al., 2017 ▶). 

**Animals and experimental procedures**

Forty healthy female Sprague-Dawley 50-55 day old rats were divided into two groups. Control group (n=10); received a single dose of DMBA solvent orally and treated group with DMBA dissolved in olive oil (n=30). DMBA-treated group received DMBA at dose of 40 mg/kg body weight by oral gavage. After tumor emergence, DMBA-treated group was randomly divided into 3 equal groups. DMBA group (group I) received distilled water (i.e. AOE solvent) by oral gavage. Group II and III (DMBA+AOE groups) received AOE 120 and 240 mg/Kg/day, respectively by oral gavage for 60 consecutive days. At the end of the experimental period, animals were euthanized by diethyl ether, blood samples were collected for evaluation of selected markers and tumor samples were dissected for further examination.

**Determination of total phenolic content**

The total phenolic content of AOE extract was determined using Folin-Ciocalteau method with some modifications. Total phenol values were expressed as mg gallic acid equivalent/g dried extract (Zhishen et al., 1999 ▶). 

**Determination of total flavonoid content**

The total flavonoid content of AOE was determined using aluminum chloride colorimetric method with slight modification. The total flavonoid values were expressed in terms of mg rutin equivalents/g dried extract (Chang et al., 2002 ▶). 

**Determination of free radical scavenging ability**

The free radical scavenging activity of AOE was determined by ABTS radical cation decolorization assay, which is based on the reduction of ABTS+• radicals by antioxidants of the plant extracts tested. Percent of inhibition was calculated as follows: %Inhibition = [(A_0_ - A_E_)/A_0_] ×100. A_0_ is the absorbance of control and A_E_ is the absorbance of the plant extract. 

The DPPH radical scavenging activity of AOE was assessed using the method proposed by Von Gadow. with little modifications. Percent of inhibition was calculated as follows: % Inhibition = [(A_0_ - A_E_)/A_0_] ×100. A_0_ is the absorbance of control and A_E_ is the absorbance of the plant extract (Von Gadow et al., 1997 ▶). 

**Determination of ferric reducing antioxidant potential (FRAP) assay**

The ferric reducing power of extract was assessed according to Benzie and Strain with some modifications. The FRAP content was expressed as µmol/L for plasma using FeSO4.7H2O solution as standard (0-1500 µmol/L). FRAP values were expressed as µM Fe (II) /mg extract (Doustimotlagh AH et al. 2014). 

**Diameter and volume of breast tumors**

The final diameter of tumors was measured using a caliper in millimeters (mm) and the final volume (cm^3^) was calculated using the formula 4/3 πr3 (r=1/2 tumor diameter (cm).

**Serum concentrations of CA**_15.3_ **and TP**_53_ **assay**

Serum concentrations of CA_15.3 _and TP_53_ were measured using micro plate double-antibody sandwich enzyme-linked immunosorbent assay (ELISA) as described in the instructions provided by manufacturer’s kits and expressed as U/ml and pg/ml, respectively (Kokhdan et al., 2018 ▶).

**MDA assay**

Serum MDA level, a lipid peroxidation end product, was assessed based on the reaction of thiobarbituric acid with MDA (Hodges et al., 1999 ▶). MDA concentration was determined based on a standard curve of 1, 1, 3, 3-tetraethoxypropane (TEP). Standard curve was made using serial dilution of TEP to yield the following test concentrations: 0, 1, 2, 2.5, 5, and 10 μM. 0.5 ml of serum or standard solutions was taken in a test tube and 2 ml of the TBA-TCA (TBA-TCA reagent: 0.375% w/v TBA, 15% w/v TCA, and 0.25 N HCl) solution were added. The mixture was heated in a water bath (90– 100◦C) for 15 min, cooled in a cold water bath for 10 min, and then centrifuged at 2000 g for 15 min. The absorbance of the solution was read spectrophotometrically at 535 nm. MDA was expressed as nmol/ml (Karami et al., 2018 ▶).

**Catalase activity assay**

CAT activity was measured by the method of Aebi (Aebi, 1984 ▶). An aliquot (5 µl) of each serum sample was added to a cuvette containing 1.995 ml of 50 mM phosphate buffered saline (pH 7.0). Reaction was started by addition of 1.0 ml of freshly prepared 30 mM H_2_O_2_. The rate of decomposition of H_2_O_2_ was measured spectrophotometrically at 240 nm. Activity of CAT was expressed as ×10^−1^ k/mg protein, where k represents the rate constant of the first order reaction of CAT or reaction rate coefficient (in units of 1/time)

**Serum calcium assay**

Serum calcium was measured by automated colorimetric methods (Baginski et al., 1973 ▶). First, 20 µl of serum was added into test cups and 20 µl of the working calcium standard (10 mg/dl) was added into another cup. Afterwards, 100 µl of *o*-cresolphthalein complexone (CPC) reagent was added to each cup as well as to a third cup for the reagent blank. Finally, the absorbance’s of samples and the standard was read against the reagent blank in a spectrophotometer set at 575 nm. 

**Immunohistochemistry analysis**

Immunohistochemical analysis was performed on 5-µm paraffin-embedded tissue section on poly-Llysine-coated glass slides. The tissue sections were deparaffinized by placing the slides in xylene for 10 min each. After gradual hydration through graded ethanol series (100, 96, 70, and 50%) for 10 min each, the slides finally were washed in distilled water and phosphate buffered saline (PBS) for 5 min. The sections were incubated with 3% H_2_O_2_ in distilled water for 15 min to quench endogenous peroxidase activity and diminish non-specific staining. The tissue section was then incubated and heated in citrate buffer (pH 6.0) for 40 min to antigen retrieval and immunolabeling. After cooling to room temperature and rinsing with PBS (3×5 min), tissue sections were incubated with 10% normal goat serum for 20 min at room temperature to block further non-specific antibody binding sites. Then, the sections were incubated with rabbit monoclonal anti-PCNA primary antibody (Abcam, UK) at a dilution of 1:100 in PBS overnight in the moist chamber at 4^o^C. Following PBS washes (2×5 min), the bound primary antibody was detected by incubation with the Envision secondary antibody conjugated with horseradish peroxidase (BioGenex, USA) for 30 min at room temperature. After washing with PBS (2×5 min), the antigen – antibody complex and peroxidase reaction was revealed by incubating with 3, 3′-diaminobenzidine tetrahydrochloride in chromogen solution (DAB, DAKO, Denmark), as the substrate of horseradish peroxidase, for 1–3 min and staining with haematoxylin. Finally, the sections were observed for brown color formation under a light microscope and the percentage of immunopositive cells was recorded. For negative control, the primary antibody was replaced with PBS (Ueda et al., 2005 ▶).

**Histopathological examination**

Mammary tissues were fixed in 10% buffered formalin, embedded in paraffin. The blocks were cut to obtain 5 µm thick sections and stained with hematoxylin–eosin. Serial paraffin sections of each tissue image were captured by light microscopy (Olympus IX71).

**Statistical analysis**

All results are expressed as mean±S.E.M. The differences between the control and treatment groups were tested by one-way analyses of variance (ANOVA) followed by the Tukey *post-hoc* test, using the SPSS 22 for windows. P-values<0.05 were considered to show significant differences.

## Results

**Total phenol, flavonoids and FRAP of AOE**

Total phenol, flavonoids and FRAP content of AOE at 1 mg/ml concentration is presented in Table 1.

**Diameter and volume of breast tumors**

**Figure 1:**
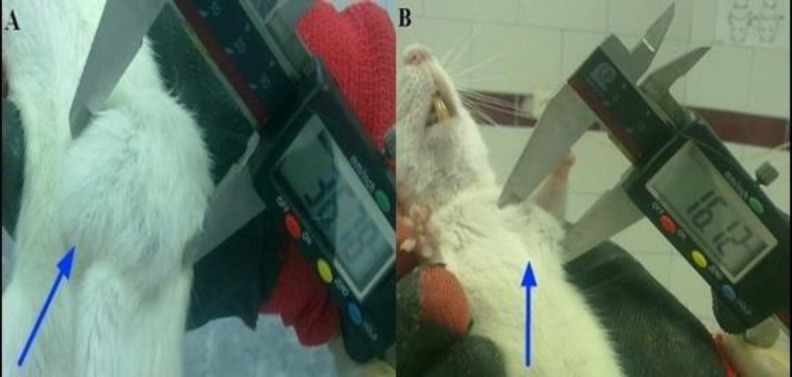
Breast tumors (A) group II (DMBA alone) and (B) group III (DMBA+120 mg/Kg AOE).

The mean values of diameter and volume of breast tumors at the end of the intervention are shown in Figure 1 and Table 2. First tumors were reported 110 days after receiving DMBA in all groups and the incidences increased during the period. The final diameter and volume of 

tumors in group III (120 mg/Kg AOE) were significantly reduced at P < 0.01 and p<0.05, respectively as compared with group II. Although these two parameters in group IV (240 mg/Kg AOE) decreased when compared with DMBA group, differences were not statistically significant.

**Effect of DMBA and AOE on serum content of CA**_15.3_

**Figure 2:**
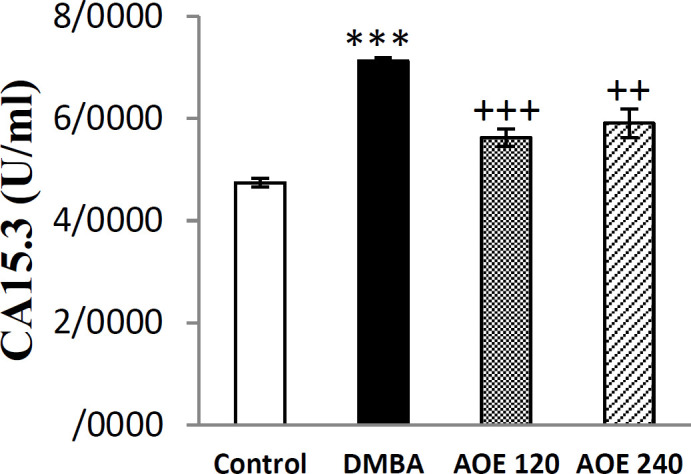
Serum levels of CA15.3 in the control, DMBA and treated groups (AOE 120 and AOE 240). Data are presented as mean±SEM. ***: p<0.001 indicates significant differences with control group, +++p<0.001, ++p<0.01 show significant differences with DMBA group

As shown in Figure 2, serum levels of CA_15.3 _in the DMBA-treated group significantly increased compared to the control group (p<0.001). Treatment with AOE at dose of 120 and 240 mg/Kg/day body weight significantly decreased the serum levels of CA_15.3 _compared to DMBA-treated group (p<0.001 and p<0.01 respectively).

**Effect of DMBA and AOE on serum content of Tp**_53_

**Figure 3:**
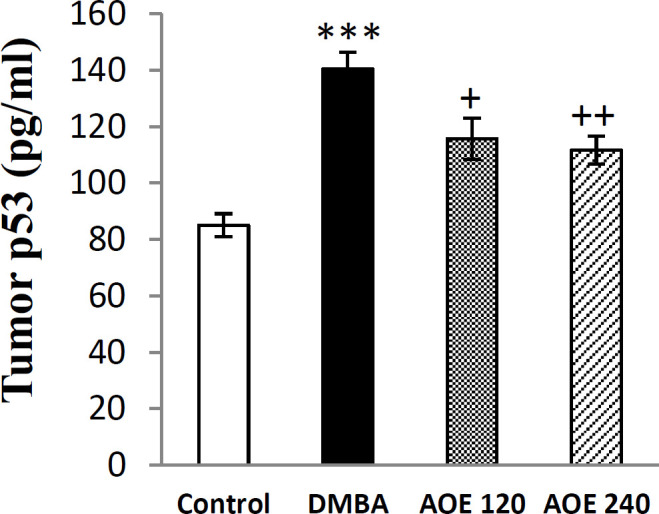
Serum levels of Tp53 in the control, DMBA and treated (AOE 120 and AOE 240) groups. Data are presented as mean±SEM, ***: p<0.001 indicates significant differences with control group, ++: p<0. 01, +: p<0.05 show significant differences with DMBA group

The serum levels of Tp53 at the end of the intervention period, are presented in Figure 3. The value of Tp53 in DMBA-treated group significantly rose when compared to the control group (p<0.001). Treatment with AOE at dose of 120 and 240 mg/Kg/day body weight significantly reduced the levels of Tp53 in a dose-dependent manner in comparison to the DMBA group (p<0.05 and p<0.01 respectively).

**Table 1:** Total phenolic and flavonoid contents, as well as ABTS, DPPH and FRAP value of the AOE

Sample	Total phenol^a^	Total flavonoid^b^	ABTS (%)	DPPH (%)	FRAP (µM/mg)
AOE	180.2±8.08	127.99±0.94	21.02±4.07	12.61±.04	805.67±0.94

Each value represents the mean±S.D. (n=3).

^a^Total phenolic content was expressed as mg gallic acid equivalents/g dried extract.

^b^Total flavonoid content was expressed as mg rutin equivalents/g dried extract.

**Table 2:** Effect of AOE on final diameter and volume of tumor in experimental animals

Particulars	DMBA	AOE120	AOE240
Tumor diameter (mm)	34.664±1.705	21.545±1.745^**^	30.96±2.4
Tumor volume (cm^3^) rat^-1^	22.384±3.34	5.325±1.27^**^	15.765±3.615

Data are presented as mean±SEM for ten animals in each group (n=10).

** p<0.001 show significant differences with DMBA group.

**Effect of DMBA and AOE on serum content of MDA**

**Figure 4:**
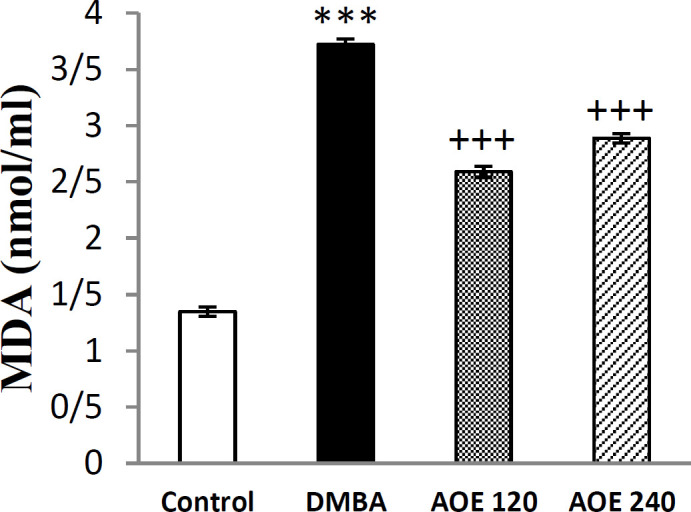
Serum levels of MDA in the control, DMBA and treated (AOE 120 and AOE 240) groups. Data are presented as mean±SEM.***p<0.001 indicates significant differences with control group, +++ p<0.001 show significant differences with DMBA group

As shown in Figure 4, the serum levels of MDA in DMBA-treated group considerably augmented in comparison to the control group (p<0.001). Oral treatment with AOE at doses of 120 and 240 mg/Kg significantly inhibited the elevation of serum MDA (p<0.01). 

**Effect of DMBA and AOE on serum catalase activity**

**Figure 5:**
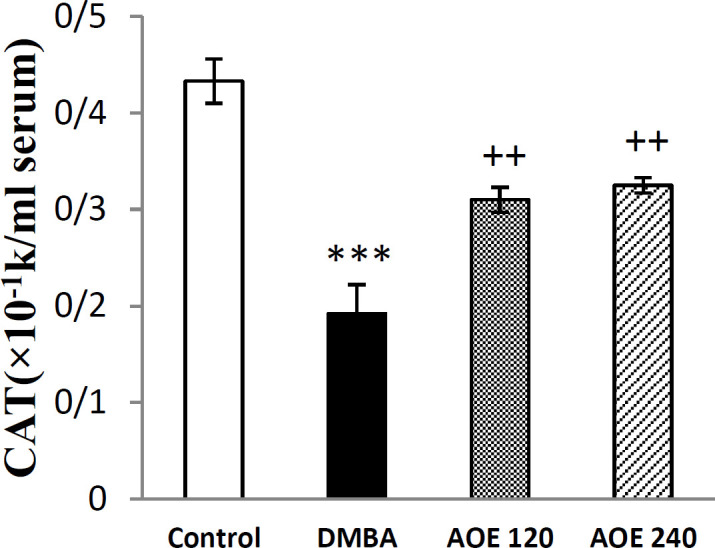
Serum levels of CAT in the control, DMBA and treated groups (AOE 120 and AOE 240). Data are presented as mean±SEM, ***p<0.001 indicates significant differences with control group, ++p<0.01 show significant differences with DMBA group

As illustrated in Figure 5, serum activity of CAT significantly declined in DMBA-treated group in comparison to the control group (p<0.001). Oral intake of AOE concurrently with DMBA at both doses of 120 and 240 mg/kg, increased the reduced activities of CAT (p<0.01 for both cases), as compared to DMBA-treated rats.

**Effect of DMBA and AOE on serum calcium content**

**Figure 6:**
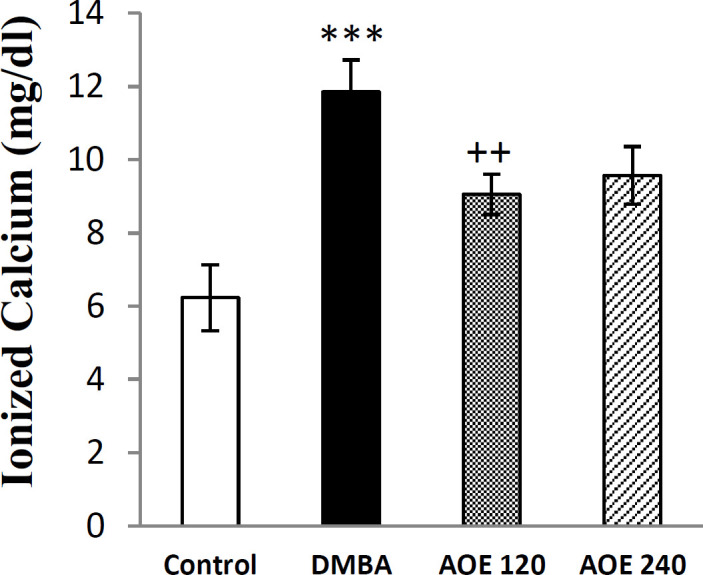
Serum levels of ionized calcium in the control, DMBA and treated groups (AOE 120 and AOE 240). Data are presented as mean±SEM. ***: p<0.001 indicates significant differences with control group, ++p<0.01 compared to DMBA group

Serum levels of Ca^2+^ at the end of the intervention period, are shown in Figure 6. Ionized calcium in the DMBA group significantly increased compared to the control group (p<0.001). AOE at both doses (120 and 240 mg/Kg) decreased the serum calcium content compared to DMBA- treated group, but only at the dose of 120, the difference was statistically significant (p<0.01).

k represents the rate constant of the first order reaction of CAT).

**Immunohistochemistry analysis**

**Figure 7:**
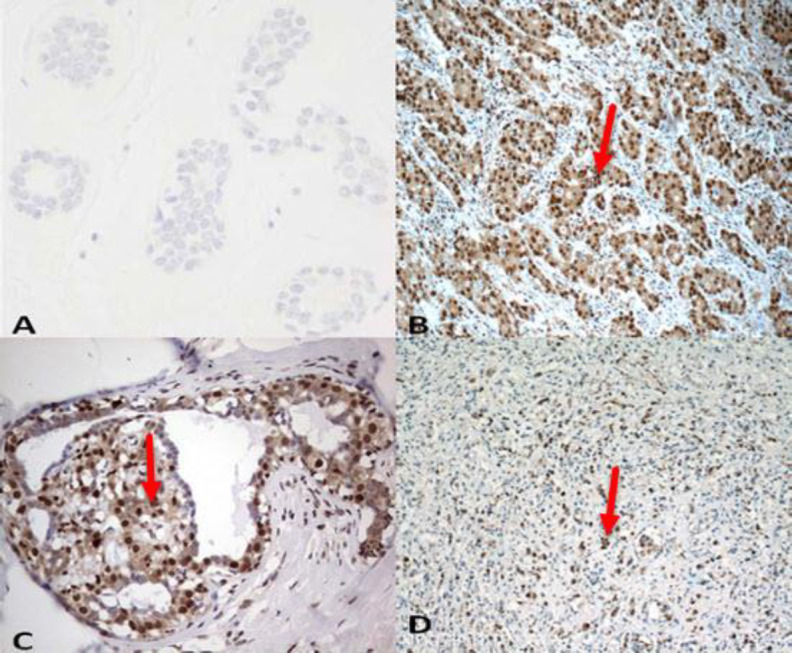
Immunohistochemical expression of PCNA in mammary tissues of rats. (A); control group, treated with olive oil (group I), (B and C); DMBA group (group II) and (D); DMBA+120 mg/kg of AOE (group III). PCNA proliferating cell nuclear antigen is a marker of cell proliferation, it is negative in control group (A) and markedly increased in neoplastic cells (Red Arrow) (B, C) but is markedly decreased after treatment with AOE, indicating the effectiveness of AOE in treatment of DMBA-induced breast cancer in rat. All images: 100X

 Effect of AOE on the expression of PCNA at the end of the intervention, is presented in Figure 7. Treatment with DMBA (40 mg/kg body weight) significantly induced the PCNA expression (80%) compared to the control group. Oral AOE (120 and 240 mg/kg body weight) given concurrently with DMBA significantly reduced PCNA expression up to 20% compared to the DMBA-treated group.

**Histopathological examination**

**Figure 8:**
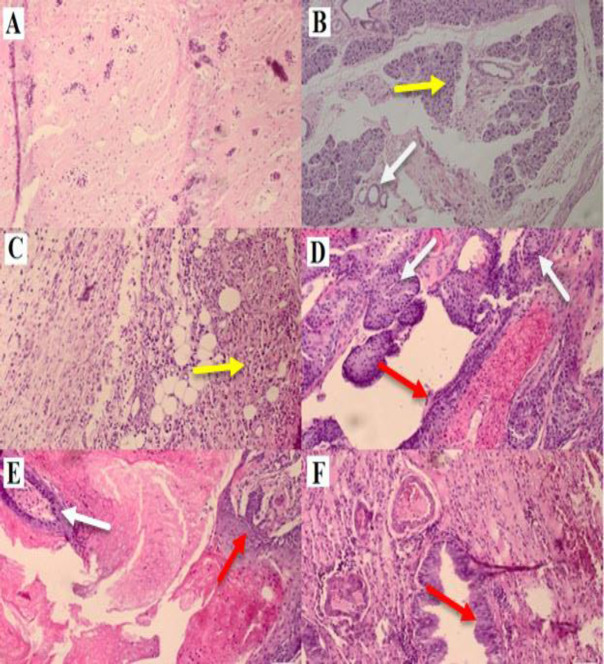
Histopathological sections of breast tissue (H&E stain) in control and experimental groups. (A); shows preserved normal ductular and alveolar structure of mammary tissue with epithelial cells of uniform appearance (control group). (B, C and D); Treatment of rats with DMBA alone (group II) caused an invasive ductal carcinoma (White Arrow) with acute inflammation, tumor cell (Yellow Arrow) and squamous differentiation, similar to cancer of skin cells with large areas of keratinization (Red Arrow). (E and F); The AOE 120 mg/Kg noticeably reduced these changes, induction of necrosis and prevention of DMBA-induced breast cancer in rats

 Histopathological examination of the tumor sections is presented in Figure 8. In the control group, histopathological evaluation of breast tissue revealed normal histological architecture with normal ductular and alveolar structure of mammary tissue with epithelial cells of uniform appearance (Figure 8.A). Treatment with DMBA alone (group II) induced an invasive ductal carcinoma with extensive areas of necrosis and acute inflammation (Figure 8B) and differentiated squamous cell carcinoma (similar to cancer of skin cells) with large areas of keratinization and inflammatory (Figure 8C and D). Co-administration of AOE (120 and 240 mg/Kg) and DMBA noticeably improved the histological changes observed in DMBA-treated group (Figure 8 E and F, respectively). 

## Discussion

Despite recent advances in the treatment of breast cancer, it is one of the leading causes of cancer-related death among women worldwide (Amin, 2009 ▶).

For the ﬁrst time, the results of the present work showed that AOE exerts a significant antitumor activity against DMBA-induced mammary tumorigenesis in female Sprague-Dawley rats.

In Chinese folk medicine, *Astragalus* has been used for more than 2,000 years. Increasing documents have found that it has promising anticancer, such as increasing the responsiveness of antitumor agents, causing cell death, and preventing cell proliferation, and antioxidant effects (Wang et al., 2015 ▶; Zhu et al., 2015 ▶; Li W et al., 2018 ▶; Park and Park, 2018 ▶; Zhou R et al., 2018 ▶).

DMBA-induced mammary gland tumor in rats is a well-known model which has been widely used for evaluation of different compounds as chemopreventive drugs for breast cancer in humans (Kelloff et al., 1995 ▶; Lai and Singh, 2006 ▶). 

DMBA carcinogenicity is associated with its oxidative metabolism leading to the formation of reactive metabolites, which bind covalently to nucleophilic sites on cellular macromolecules eliciting cancerous responses (Ojeswi et al., 2010). 

 The present data showed that* A. ovinus* effectively reduced diameter and volume of the tumor in tumor-bearing rats. In order to reveal some possible mechanisms involved in anticancer effect of AOE, we first investigated some events that lead to cell cancer development (Figure 1).

Cell proliferation is regulated by multiple mechanisms. PCNA, a nuclear protein in proliferating cells, is essential for replication and serves as a cell proliferation marker (Amin, 2009 ▶). PCNA overexpression was reported in a variety of human tumors and carcinoma induced by DMBA (Subapriya et al., 2006 ▶). In this study, PCNA over-expression in breast tissues of DMBA-treated group indicated an increased cell proliferation and is consistent with previous study (Vinothini et al., 2009 ▶). Immunohistochemical staining showed that the treatment of rats with AOE reduced the expression of PCNA that suggests anti-proliferative mechanisms involved in the observed chemopreventive action of the extract (Figure 7).

Serum marker CA_15.3_, which is used widely for breast cancer diagnosis, is recommended for evaluation of metastatic breast cancer response to treatment and monitoring (Duffy, 2006 ▶; Quaranta et al., 2007 ▶). It was shown that high levels of CA_15.3 _in serum suggest poor response to immunotherapy (Martin et al., 2006 ▶). As shown, oral treatment of rats with AOE significantly reduced the serum level of CA_15.3 _compared to DMBA-treated group (Figure 2). These results were in accordance to the reports suggesting the chemotherapeutic effect of Octyl gallate and gallic acid, plant derived compounds, on DMBA-induced mammary tumor in rats (Rajalakshmi and Sales, 2015 ▶).

Numerous studies demonstrated that p53 status is an important determinant of the response of tumors to anti-neoplastic agent (Manna et al., 2011 ▶). Mutations in p53 were reported to occur in 40% of all human tumors. Mutant p53 acts as an oncogene, loses its ability to act as a tumor suppressor, and enhances cell proliferation. Overexpression of mutant p53 may increase genetic instability by facilitating cell proliferation (Subapriya et al., 2006 ▶). Our results showed that serum levels of mutant p53 in DMBA-treated group are significantly increased in comparison to the control group. Oral treatment with AOE significantly decreased the serum levels of mutant P53 compared to the DMBA group (Figure 3).

It was reported that antioxidants act as one of the primary line of body defense against free radicals (Mansourian et al., 2018 ▶) and suggested that antioxidant agents protect cells from injury induced by unstable free radicals and reduce the risk of oxidative damage during carcinogenesis (Anbuselvam et al,. 2007 ▶; Sadeghi et al., 2019 ▶), therefore, it is possible that use of antioxidants can be helpful in inhibiting cancer development or treatment.

In this study, first, in vitro antioxidant activity of AOE was investigated using FRAP, DPPH and ABTS assays. The FRAP assay, is presented as a novel method for assessing antioxidant power and serve as an important indicator of potential antioxidant activity of different compounds (Azarmehr et al., 2019 ▶). In this study, AOE showed a good concentration-dependent activity in the FRAP test (Table 1). These results are similar to those of Li et al. (Li et al., 2009 ▶).

Furthermore, DPPH and ABTS radical scavenging assays are general spectrophotometric procedures for assessment of antioxidant capacities of components (Schaich et al. 2015 ▶). In the present study (Table 1), AOE showed pronounced DPPH and ABTS radical scavenging activity, and it was almost as effective as ascorbic acid at similar concentrations which was consistent with the findings of Li et al. (Li et al., 2009 ▶). 

For evaluation of the in vivo antioxidant activity of AOE, we measured the CAT enzyme activity and level of MDA, as a lipid peroxidation marker (Arya et al., 2019 ▶) in the serum samples. DMDA increased the serum level of MDA in the DMBA-treated group compared to the control group. Oral administration of AOE to rats exposed to DMBA, decreased the levels of MDA and improved antioxidant CAT activity (Figures 4 and 5).

It is important to mention that physiological concentrations of extracellular Ca^2+^ down-regulated cell proliferation and invasion (Liu et al., 2009 ▶). In experimental studies, the decreased level of extracellular Ca^2+^ reduces cell proliferation and induces the differentiation of breast cells (Cui and Rohan, 2006 ▶). Calcium is a crucial second messenger participating in cellular functions such as cell differentiation, proliferation, apoptosis modulating enzyme secretion and gene activation (Almquist et al., 2010 ▶). Furthermore, along with other proliferative markers such as PCNA, serum calcium level was also 

measured in the present study. The data revealed that DMBA administration increased the serum calcium in the DMBA-treated group compared to the control group. This alteration was significantly inhibited by AOE (Figure 6), but determining the exact mechanism of calcium increase requires further studies.

As indicated in the results, AOE contains significant amounts of phenolic and flavonoids compounds. Flavonoids, a group of natural components are found in fruits, vegetables and herbal medicine. These natural substances are famous for their valuable effects on human health. It has been demonstrated that flavonoids have anti-oxidative, anti-inflammatory, anti-tumor activities that were attributed to their capacity to regulate main cellular enzyme function (Khan et al., 2012 ▶). Furthermore, phenolic substances are large heterogeneous components of secondary herbal metabolites that have been broadly found in herbs ( Arumugam , et al. 2019 ▶). These natural substances rich in antioxidants, are of crucial value for researchers (Padmavathi et al., 2006 ▶; Yazdanparast and Ardestani, 2007 ▶).

Breast cancer is associated with a change in the activity of antioxidant enzymes and cellular redox conditions, excessive cell proliferation, dysregulation of cell differentiation and insufficient apoptosis (Sahin et al., 2011 ▶). Furthermore, oxidants play an important role in multi-stage carcinogenesis from start to development (Nishigori, 2006 ▶), therefore, antioxidant and antitumor effects of the AOE are possibly due to the presence of phenolic and flavonoid compounds. In this regard, there are some reports that the anticancer effects, scavenging of DPPH radicals and increasing of SOD and CAT activities are attributed to the phenolic and flavonoid compounds (Xu et al., 2014 ▶; Zhu et al., 2015 ▶). In another study, the antioxidant and hepatoprotective properties of *Astragalus* against CCl_4_-induced liver damage were refered to its antioxidants including reducing lipid peroxidation and increasing the activity SOD and total antioxidant capacity (Jia et al., 2012 ▶).

The effect of 120 mg/Kg dose in some indices was more than 240 dose. Therefore, one possibility is that some of the active constituent(s) of *A. ovinus* at high doses (240 mg/kg) may promote tumor growth. The similar results have been reported in other studies regarding medicinal plant (Sadeghi H., 2014 ▶; Maleki N., 2001 ▶). It is important to note that the main reason for this finding requires further research.

The results of present confirmed that AEO exhibits a significant inhibitory effect on the DMBA-induced breast cancer in rats and support the potential usage of this plant in the management of breast cancer. The precise mechanism of this effect is not clear; however, antioxidant properties and inhibitory effects of the plant on the PCNA, and p53 pathways (apoptosis) may be important in its chemopreventive properties. 
